# Involvement of TRPA1 in Necrosis of Melanoma Cells via Phospholipase D1

**DOI:** 10.3390/cells15090760

**Published:** 2026-04-23

**Authors:** Rei Nakano, Manami Kuji, Mana Sugimura, Naoya Yachiku, Nanako Kitanaka, Taku Kitanaka, Yoko Suwabe, Atsuto Naruke, Junichi Nunomura, Masami Uechi, Tomohiro Nakayama, Hiroshi Sugiya

**Affiliations:** 1 Laboratory for Mucosal Immunity, RIKEN Center for Integrative Medical Sciences (IMS), 1-7-22 Suehiro-cho, Tsurumi-ku, Yokohama 230-0045, Kanagawa, Japan; 2 Laboratory of Veterinary Radiotherapy, College of Bioresource Sciences, Nihon University, 1866 Kameino, Fujisawa 252-0880, Kanagawa, Japan; 3 Japan Animal Specialty Medical Institute, Tsuzuki-ku, Yokohama 224-0001, Kanagawa, Japan

**Keywords:** melanoma, cell death, Ca^2+^ influx, transient receptor potential ankyrin 1, phosphatidylcholine-specific phospholipase D1

## Abstract

The tumor microenvironment, including extracellular pH (pH*e*), has emerged as a key regulator of tumor cellular function. Although extracellular acidification sensing and function are well established, the effect of extracellular alkalinization on cellular functioning remains unclear. Here, we report that transient receptor potential ankyrin 1 (TRPA1) functions as an alkaline sensor and mediator of cell death in melanoma cells. Exposure to alkaline pH*e* (8.1) or allyl isothiocyanate (AITC), a TRPA1 agonist, significantly reduced melanoma cell viability. We found that cell death was propidium iodide-positive and annexin V-negative, suggesting that pH*e* or AITC treatment induced necrosis rather than apoptosis. TRPA1 activation induced sustained Ca^2+^ influx, which was suppressed by either extracellular Ca^2+^ removal or treatment with the TRPA1 inhibitor, HC-030031, both of which attenuated cell death. Pharmacological screening has identified phosphatidylcholine-specific phospholipase D1 (PLD1) as a positive regulator of cell death. We confirmed that transfection with PLD1 siRNA significantly reduced AITC-induced cell death, whereas PLD2, PLD3, and NAPE-PLD siRNAs had no effect. These observations suggest that the vulnerability of melanoma cells to alkaline pH*e* is mediated by activation of the TRPA1-PLD1 axis. Thus, TRPA1 and PLD1 are potential targets for therapeutic intervention in melanoma.

## 1. Introduction

Previous studies have suggested that cells possess several sensors for monitoring extracellular pH (pH*e*) [1,2,3,4]. It is well-accepted that acidic pH monitoring can be attributed to several ion channels; however, alkaline pH monitoring mechanisms have not yet been characterized. The extracellular alkaline pH sensor transient receptor potential ankyrin 1 (TRPA1) belongs to the TRP superfamily. TRPA1 is expressed in neurons, keratinocytes, and melanocytes. Cellular responses to alkaline environments via TRPA1 have been investigated in neurons, odontoblasts, and cementoblasts [5,6,7]. The Cancer Genome Atlas (TCGA) datasets showed that TRPA1 is highly upregulated in diverse cancer types [8]. In melanoma, TRPA1 expression has also been reported [9,10]. However, the role of TRPA1 in melanoma remains poorly understood. Therefore, understanding whether TRPA1 induces or prevents cell death in melanoma is mandatory to design alternative therapies melanoma resistant to conventional therapy.

Cell death is classified as accidental cell death (ACD) or regulated cell death (RCD). ACD is a biologically uncontrolled process, whereas RCD involves tightly structured signaling cascades and molecularly defined effector mechanisms. A growing number of non-apoptotic forms of RCD have been identified and are implicated in pathophysiological events. RCD can be classified into multiple subclasses based on their molecular characteristics [11], some of which have clear physiological functions (e.g., necroptosis and pyroptosis), whereas others (e.g., ferroptosis, entotic cell death, NETotic cell death, parthanatos, lysosome-dependent cell death, autophagy-dependent cell death, alkaliptosis, and oxeiptosis) are less studied and are limited as cellular responses to specific toxins. The importance of genetic or pharmacological interventions for disrupting the lethal cascade triggered by external stimuli has been increasingly recognized.

Here, we report that alkaline pH and allyl isothiocyanate (AITC) induce melanoma cell death via TRPA1 activation. Pharmacological screening and genetic deletion analysis revealed that phosphatidylcholine-specific phospholipase D1 (PLD1), an important lipid signaling enzyme, plays a crucial role in TRPA1-induced cell death.

## 2. Materials and Methods

### 2.1. Cell Culture

Canine melanoma cells (MCM-N1 cell line; 13-year-old male dog; chromosome number, 2n = 74) obtained from DS Pharma Biomedical Co., Ltd. (Osaka, Japan) were cultured in a 100 mm dish containing DMEM-LG (FUJIFILM Wako Chemical Corp., Osaka, Japan) supplemented with 10% fetal bovine serum (FBS), 100 unit/mL penicillin, and 100 μg/mL streptomycin and maintained at 37 °C in a humidified incubator with 5% CO_2_, as previously described [1,12,13]. The cells were cultured under extracellular pH 5.4, 7.4, and 8.1 with 20 mM N-2-hydroxyethylpiperazine-N′-2-ethanesulfonic acid (HEPES); cell growth was assessed by the MTT assay. For specific activation of TRPA1, the cells were treated with AITC (2 μM).

### 2.2. Real-Time Quantitative PCR (RT-qPCR)

RT-qPCR was performed as previously described [14,15,16,17]. Canine melanoma cell samples were collected using TRIzol reagent (Life Technologies, Carlsbad, CA, USA). First-strand cDNA was synthesized from 500 ng of extracted total RNA using PrimeScript RT Master Mix (TaKaRa Bio, Inc., Kusatsu, Shiga, Japan). RT-qPCR was performed in a total reaction volume of 25 μL comprising 2 μL of the first-strand cDNA, 12.5 μL SYBR Premix Ex Taq II (TaKaRa Bio, Inc.), and 0.4 μM forward and reverse primers specific for canine PLD1, 2, 3 and NAPE-PLD. Primers for the TATA box-binding protein (TBP), a housekeeping protein, were used as a control. Table 1 shows the primer sequences used. PCR was performed with the following cycling conditions using the Thermal Cycler Dice Real Time System II (TaKaRa Bio, Inc.): 1 cycle of denaturation at 95 °C for 30 s, 40 cycles of denaturation at 95 °C for 5 s, and annealing/extension at 60 °C for 30 s. Relative mRNA levels were normalized to the endogenous reference gene TBP and quantified using the ΔΔCt method. The cDNA from untreated canine melanoma cells was defined as the calibration standard (relative expression = 1.0), and the second derivative maximum method was employed for Ct determination via the real-time RT-PCR analysis software for the Thermal Cycler Dice Real Time System II (v5.11c, TaKaRa Bio, Inc.).

### 2.3. Transfection of siRNA

The lipofection of siRNA was conducted as previously described [14,15,16,17,18]. Canine melanoma cells were seeded in 35 mm or 100 mm dishes at a density of 1 × 10^5^ or 5 × 10^5^ cells, respectively. To transfect siRNA, the cells were incubated in Opti-MEM (Life Technologies) containing 5 μL/mL Lipofectamine 2000 (Life Technologies) and 100 nM siRNAs for 6 h. After transfection, the medium was changed to DMEM-LG with 10% FBS, and the cultures were retained at 37 °C in a humidified incubator with 5% CO_2_ for 5 days. The siRNA sequences are listed in Table 2. The efficiency of the siRNAs was assessed by RT-qPCR.

### 2.4. MTT Assay

Cells were seeded at a density of 3000 cells/200 μL in each well of a 96-well plate. MTT assay reagent was dissolved in phosphate-buffered saline (PBS) at a concentration of 5 mg/mL, and 20 μL of the reagent was incubated with cells for 1 h in an incubator with 5% CO_2_ at 37 °C [1]. Following incubation, PBS (100 μL) was added to each well. After 1 min, the supernatant was discarded and the MTT formazan crystals were dissolved in 200 μL of 0.04 M hydrochloric acid in 2-propanol. The optical density (O.D.) was determined using a microplate reader (Fluoroskan Ascent FL, Thermo Fisher Scientific K.K., Yokohama, Kanagawa, Japan) at 570 nm wavelength.

### 2.5. Annexin V and Propidium Iodide Staining Following Flow Cytometry Analysis

Annexin V and propidium iodide staining was performed with ApoAlert Annexin V-FITC Apoptosis Kit (TaKaRa Bio, Inc.). Cells (1 × 10^6^/100 mm dish) were incubated with 0.25 *w*/*v*% Trypsin-1 mmol/L EDTA (FUJIFILM Wako Chemical Corp.) for 2 min at 37 °C in a humidified incubator with 5% CO_2_. The collected cells were centrifuged at 300× *g* for 1 min and suspended with 800 μL of binding buffer in 5 mL round-bottom tubes. The cells were incubated with 0.5 μg/mL Annexin V and 2.5 μg/mL propidium iodide for 15 min at 25 °C in the dark. Data were obtained by recording 10,000 events using BD FACSAria II (Becton, Dickinson and Company, Franklin Lakes, NJ, USA) and analysis was performed using Flowing Software 2.5.1 (https://bioscience.fi/services/cell-imaging/flowing-software/, accessed on 8 November 2021).

### 2.6. Inhibitor Screening Assay

The cells were pretreated with the inhibitors listed in Table 3. After the 1 h of pretreatment, the cells were treated with AITC (2 μM) followed by Annexin V and propidium iodide staining.

### 2.7. Ca^2+^ Imaging

Cells were seeded on 35 mm glass-based dishes at a density of 4000 cells/cm^2^. The cells were incubated with 4 µM Fluo-3-AM (Dojindo Lab., Kamimashiki-gun, Kumamoto, Japan) for 30 min at 37 °C in the dark [14,19,20,21]. Following incubation, the cells were washed twice with PBS. After washing, the culture medium was replaced with a Ca^2+^ imaging buffer (120 mM NaCl, 5 mM KCl, 0.96 mM NaH_2_PO_4_, 1 mM MgCl_2_, 11.1 mM glucose, 1 mM CaCl_2_, 1 mg/mL bovine serum albumin, and 10 mM HEPES; pH 7.4). Glass-based dishes with fluorescent dye-loaded cells were placed at 25 °C on a confocal laser scanning microscope (LSM510, Carl Zeiss AG, Oberkochen, Germany). The frames in the time-lapse sequence were captured every 1 s. After baseline images were acquired, the cells were stimulated with 2 μM AITC. To examine the effects of TRPA1, HC-030031 (50 μM) was used as an inhibitor of TRPA1. After pretreatment for 5 min with the TRPA1 inhibitor, the cells were stimulated with 2 μM AITC. Relative changes in intracellular Ca^2+^ concentrations over time were expressed as relative changes in baseline fluorescence.

### 2.8. In Silico Analysis of TRPA1 Expression in Human Melanoma Cells

We performed in silico analyses using the Gene Expression Profiling Interactive Analysis 3 (GEPIA3) database (https://gepia3.bioinfoliu.com, accessed on 30 March 2026) [22], which integrates data from TCGA and the Genotype-Tissue Expression (GTEx) projects. The mRNA expression levels of TRPA1 in human melanoma (TCGA-SKCM) were compared with those in normal skin using Expression DIY module. Data were normalized as Transcripts Per Million (TPM) and log_2_ (TPM + 1) transformed for differential expression analysis, with a log_2_ fold change cutoff of 1.0 and a q-value cutoff of 0.05.

### 2.9. Statistical Analysis

Statistical analysis was performed using StatMate IV (v.4.01, ATMS, Tokyo, Japan). Data from the time-course study and other experiments were analyzed using a two-way analysis of variance and Student’s two-tailed *t*-test or one-way analysis of variance, respectively. Data are presented as the mean ± standard error of measurement (SEM).

## 3. Results

### 3.1. TRPA1 Activation Induces Cell Death in Melanoma Cells

**Figure 1:**
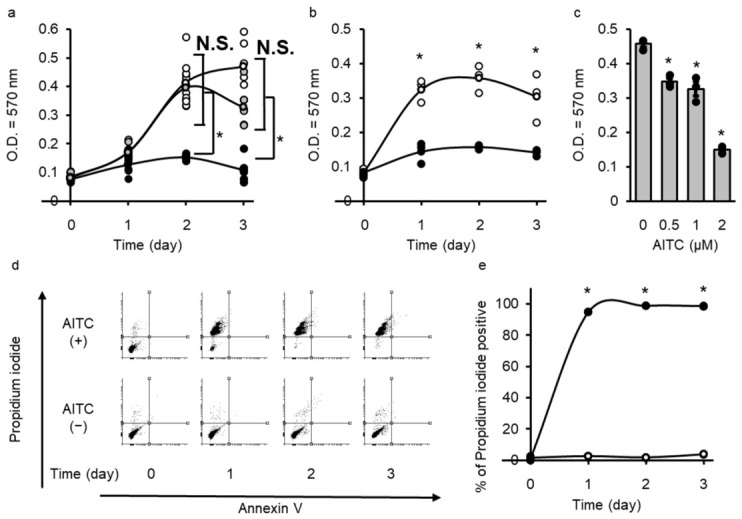
Transient receptor potential ankyrin 1 (TRPA1) activation induces necrosis in melanoma cells. (**a**) Effect of extracellular pH on melanoma cell viability. The cells were cultured at pH 5.4 (gray circles), 7.4 (open circles), and 8.1 (closed circles) for 3 days. Cell viability was assessed by MTT assay. (**b**) The cells were cultured with (closed circle) or without (open circle) 2 μM allyl isothiocyanate (AITC). Cell viability was assessed by MTT assay. (**c**) Dose-dependent effect of AITC on melanoma cell viability. The cells were treated with the indicated concentrations of AITC for 3 days, and cell viability was assessed by MTT assay. (**d**,**e**) Apoptosis (Annexin V) and necrosis (propidium iodide) in AITC-treated cells were detected using flow cytometry. Representative images of the flow cytometry analysis are shown (**d**), and the necrosis rate (positivity for propidium iodide) was calculated in the presence (closed circle) and absence (opened circle) of AITC. (**e**). Data are presented as means ± SEM of three independent experiments and compared using two-way ANOVA with Tukey’s honestly significant difference test for post hoc multiple comparison test (**a**–**c**,**e**). N.S., not significant, * *p* < 0.05, compared with pH 7.4 (**a**), untreated cells (**b**,**e**) or 0 μM (**c**).

The extracellular pH is important for the growth of several types of cancer cells. Thus, we examined the growth of melanoma cells under a series of extracellular pH conditions (pH 5.4, 7.4, and 8.1) using MTT assay. The cells were incubated with pH 8.1 for 3 days which led to significant attenuation of their growth (Figure 1a).

Members of the transient receptor potential (TRP) channel family are ubiquitously expressed in several cell types and tissues. They exhibit diverse physiological functions, and many of them play important roles in the perception of a wide spectrum of physical and chemical stimuli (i.e., mechanical stress, temperature, and pH) [23]. TRPA1 is predominantly expressed in sensory neurons, odontoblasts, and cementoblasts and is a central molecule in sensing chemical and physical stimuli [6,7,9]. It has been reported that an alkaline extracellular pH, or consumption of certain species or herbal medicines, such as AITC, can induce the activation of TRPA1 [5,24]. To confirm the translational relevance, we analyzed TRPA1 expression in public datasets of human melanoma (Figure S1). Our in silico analysis revealed that the expression of TRPA1 increased in melanoma compared with normal tissue. These observations suggest that TRPA1 remains constitutively expressed in melanoma, suggesting a stable molecular target for therapeutic intervention. However, the function of TRPA1 in melanoma cells is still unclear. Since canine melanoma model is a clinically relevant spontaneous model that shares several features with human non-UV-induced melanomas (i.e., aggressive local invasion and a high metastatic rate), we investigated whether TRPA1 activation inhibits the growth of melanoma cells using a TRPA1 activator AITC. The inhibition of melanoma growth was observed when the cells were treated with 2 μM AITC for three days (Figure 1b). When the cells were incubated with 0 to 2 μM AITC, a dose-dependent decrease in their growth was observed (Figure 1c).

The exposure of the outer plasma membrane to phosphatidylserine, which is detected by Annexin V staining, is a feature of apoptosis, whereas the loss of integrity of the plasma and nuclear membranes, detected by propidium iodide (PI) staining, is characteristic of necrosis [25]. A time-dependent increase in PI-positive cells was observed in AITC-treated cells, whereas Annexin V-positive cells were not detected (Figure 1d,e). These observations suggest that both alkaline pH and AITC mediated cell death in melanoma cells. We termed this TRPA1-induced necrosis-like cell death TRPAptosis. Our functional analysis strongly suggests that melanomas are hypersensitive to TRPA1 activation and subsequent TRPAptosis triggered by potent agonists or environmental alkalinization. Consequently, our strategy of over-activating TRPA1 specifically exploits this intrinsic ionic fragility to selectively eliminate TRPA1-high melanoma cells.

### 3.2. The Involvement of TRPA1-Induced Ca^2+^ Influx in Cell Death of Melanoma Cells

**Figure 2:**
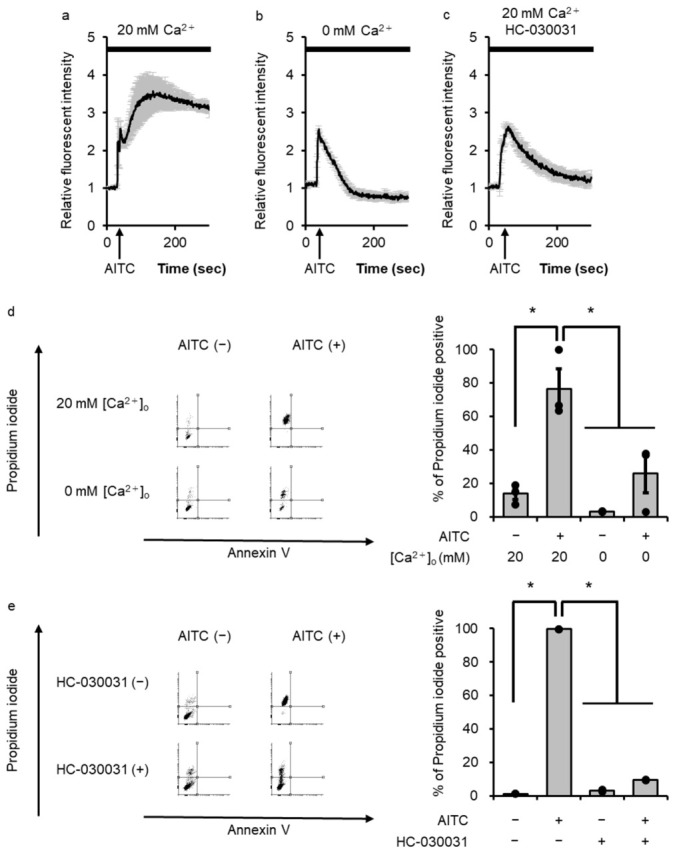
Transient receptor potential ankyrin 1 (TRPA1)-induced Ca^2+^ influx during melanoma cell death (**a**,**b**) The effect of allyl isothiocyanate (AITC) (2 μM) on [Ca^2+^]_i_ of the melanoma cells in the presence (**a**) or absence (**b**) of [Ca^2+^]_o_. (**c**) The effect of AITC (2 μM) on [Ca^2+^]_i_ of the melanoma cells in the presence of HC-030031 (50 μM). (**d**) The effect of [Ca^2+^]_o_ on AITC-induced necrosis in the melanoma cells. Representative images of the flow cytometry analysis are shown in left panel, and the necrosis rate (positivity of propidium iodide) was calculated (right panel). (**e**) Effects of HC-030031 on AITC-induced necrosis in the melanoma cells. Representative images of flow cytometry analysis are shown in left panel, and the necrosis rate (positivity for propidium iodide) was calculated (right panel). Data are presented as means ± SEM of three independent experiments and compared using one-way ANOVA with Tukey’s honestly significant difference test for post hoc multiple comparison test ((**d**,**e**) right panel). * *p* < 0.05.

TRPA1 has been reported to induce an increase in intracellular Ca^2+^ concentrations ([Ca^2+^]_i_) [5,24]. Next, we investigated the contribution of Ca^2+^ mobilization to TRPAptosis in melanoma cells. AITC treatment induced a sustained increase in [Ca^2+^]_i_ in the presence of extracellular Ca^2+^ ([Ca^2+^]_o_) (Figure 2a). To confirm the involvement of Ca^2+^ influx for the mechanism of TRPA1-induced cell death. In this study, we applied extracellular Ca^2+^-free conditions to block Ca^2+^ influx. AITC failed to induce necrotic cell death in the absence of [Ca^2+^]_o_ (Figure 2b). The TRPA1 inhibitor, HC-030031, also reduced the AITC-induced increase in [Ca^2+^]_i_ (Figure 2c). Therefore, it is conceivable that AITC promotes Ca^2+^ influx via TRPA1. We confirmed that the number of AITC-induced PI+ cells was reduced in the absence of [Ca^2+^]_o_ (Figure 2d) and in the presence of TRPA1 inhibitor (Figure 2e). These observations suggest that TRPA1-induced Ca^2+^ influx contributes to TRPAptosis in melanoma cells.

### 3.3. The Involvement of Phospholipase D (PLD) in TRPA1-Induced Cell Death in Melanoma Cells

**Figure 3:**
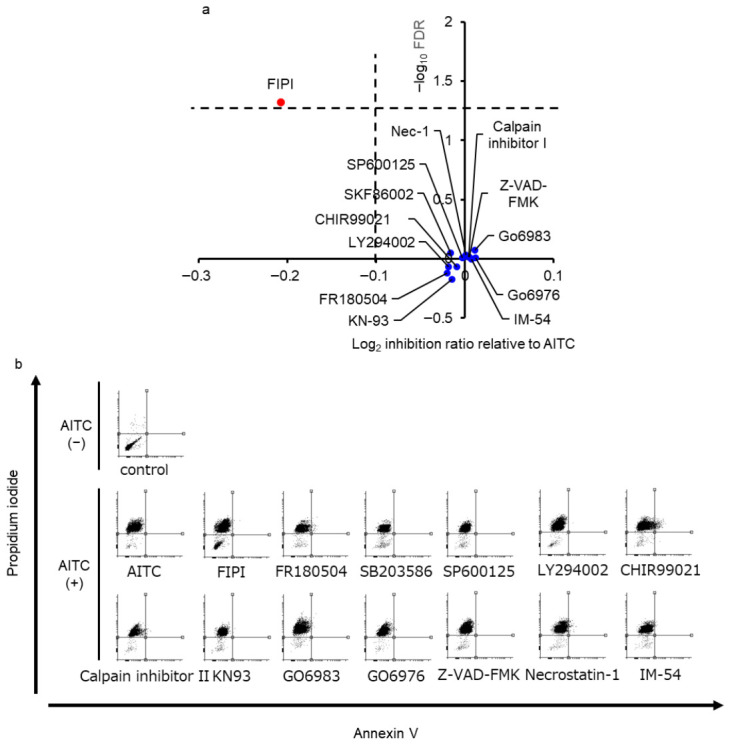
Identification of phospholipase D (PLD) as a regulator of allyl isothiocyanate (AITC)-induced necrosis in melanoma cells in the melanoma cells using an inhibitor screening assay. (**a**,**b**) Effect of inhibitors on AITC-induced necrosis in the melanoma cells. The volcano plot was constructed (**a**), and representative images of flow cytometry analysis are summarized (**b**). The PLD inhibitor, FIPI, significantly attenuated AITC-induced necrosis. The horizontal dotted line indicates the Bonferroni multiple comparison test-corrected threshold (−log_10_ FDR = 1.3) for statistical significance. The vertical dotted line indicates the threshold for the inhibition ratio of AITC-induced necrosis (log_2_ inhibition ratio = −0.1).

Cell death has been classified into two major subtypes: regulated cell death (RCD) and accidental cell death (ACD). RCD is modified by intrinsic and extrinsic signaling cues, whereas ACD is a biologically uncontrolled process [11]. Several cell death effector proteins (such as caspases, MLKL) and cellular signalings regulate specific types of RCD, all of which are inhibited by rescue compounds [11,26,27,28,29,30,31,32,33]. To classify the type of cell death of TRPAptosis, we investigated whether inhibitors of established cell death molecules attenuated TRPAptosis (Figure 3a,b). The pan-caspase inhibitor Z-VAD-FMK, which targets the apoptotic pathway failed to attenuate the cell death induced by AITC. The necroptosis inhibitors Necrostatin-1 and IM-54, which inhibit RIPK1/MLKL and mitochondrial oxidative stress-induced death, respectively [34,35], also failed to suppress AITC-induced cell death. The lack of sensitivity to these inhibitors suggests that TRPAptosis is a distinct form of necrotic cell death triggered by acute calcium overload rather than the aforementioned RCD.

Then, we performed inhibitor screening assay to identify the upstream regulator for TRPAptosis. Since TRPA1 activation leads to a sustained increase in [Ca^2+^]_i_, we selected inhibitors targeting Ca^2+^-dependent signaling molecules (e.g., calpain inhibitor II for calpain, KN-93 for CaMKII, Go6983 for pan-PKC, Go6976 for classical PKC and FIPI for PLD). Additionally, we screened major MAPK and survival pathways (FR180204 for ERK, SKF86002 for p38 MAPK, SP600125 for JNK, LY294002 for PI3K, and CHIR99021 for GSK-3) that are frequently trans-activated in melanoma and Ca^2+^ signaling. In this study, we revealed that the PLD inhibitor FIPI significantly attenuated TRPAptosis (Figure 3a,b; −0.1 > log_2_ inhibition ratio, 1.3 < −log_10_FDR). However, none of the other inhibitors attenuated TRPAptosis, suggesting that the TRPA1-mediated Ca^2+^ increase may activate PLD1 through a non-canonical mechanism. PLD hydrolyzes phospholipids to produce phosphatidic acid (PA), and the addition of PA induces calcium mobilization, suggesting crosstalk between PLD and Ca^2+^ signaling [27]. Collectively, our results suggest that PLD plays an important role in TRPAptosis.

### 3.4. PLD1 Contributes to TRPA1-Induced Necrosis in Melanoma Cells

**Figure 4:**
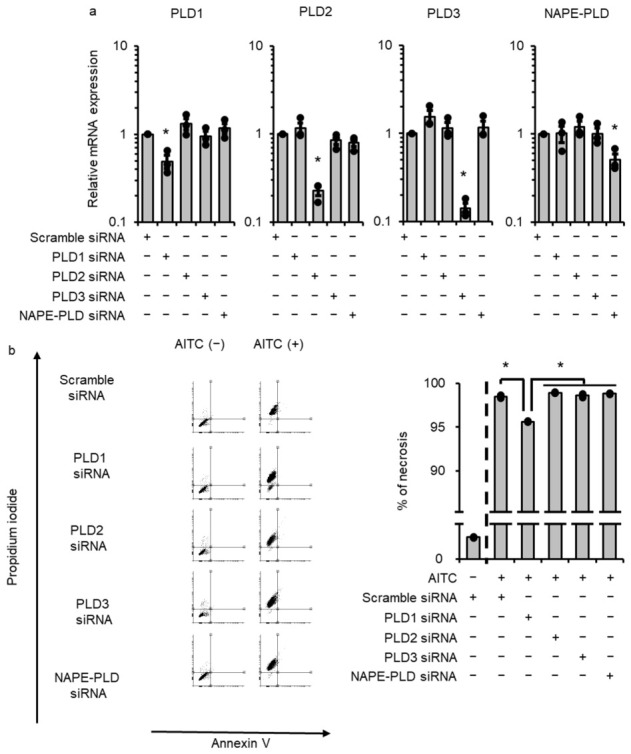
Phospholipase D1 (PLD1) contributes to the allyl isothiocyanate (AITC)-induced necrosis of melanoma cells. (**a**) Expression of PLD1, 2, 3, and NAPE-PLD mRNAs in cells transfected with specific siRNAs for PLD1, 2, 3, and NAPE-PLD, respectively. (**b**) AITC-induced necrosis was attenuated in PLD1-depleted cells, but not in cells transfected with PLD2, 3, and NAPE-PLD. Data are presented as means ± SEM of three independent experiments and compared using one-way ANOVA with Tukey’s honestly significant difference test for post hoc multiple comparison test ((**a**,**b**) right panel). * *p* < 0.05.

To confirm which PLD isoenzyme mediates TRPAptosis, we depleted the expression of each PLD isoenzyme by siRNA transfection. The following PLD isoenzymes have been reported: PLD1, PLD2, PLD3, and NAPE-PLD [36]. In melanoma cells, all the PLD isoenzymes were expressed, and the mRNA expression of each isoenzyme was attenuated in cells transfected with specific siRNAs for PLD1, 2, 3, and NAPE-PLD, whereas the mRNA expression remained stable in cells transfected with scramble siRNA (control) (Figure 4a). In PLD1-depleted cells, appearance of AITC-induced PI+ cells was significantly inhibited, whereas no attenuation of necrosis was observed in cells transfected with siRNA for PLD2, 3, NAPE-PLD, and scramble (Figure 4b). We confirmed that PLD1-specific siRNA attenuated the protein expression of PLD1 (Figure S2). These results suggest that PLD1 plays a crucial role in TRPAptosis in melanomas.

## 4. Discussion

In this study, we demonstrated that the activation of TRPA1 by alkaline extracellular pH and the TRPA1 agonist AITC induced necrosis-like cell death through Ca^2+^ influx. We termed this TRPA1-induced cell death “TRPAptosis”.

Our findings revealed a novel link between pH*e* and TRPA1 activity in melanoma cells. The tumor microenvironment (TME) is characterized by extracellular acidification owing to enhanced glycolysis and lactate production. In our study, exposure of melanoma cells to an alkaline environment (pH 8.1) induced significant cell death. In a previous study, the neutralization of extracellular acidification following oral NaHCO_3_ intake reduced the formation of a hepatic metastasis model by intrasplenic injection of a human breast cancer cell line (MDA-MB-231), which supports our notion [37]. As an alkaline pH has been reported to activate TRPA1 [5,24], our results suggest that pH*e* fluctuations in the TME trigger cell death through TRPA1. This indicates a previously unrecognized role of tumor alkalinization in modulating cancer cell viability through ion channel signaling. Alkalinization is a promising approach for acid-adapted cancer cells; however, the complexity of the in vivo TME—specifically systemic pH homeostasis and interstitial fluid pressure—presents major hurdles. To overcome these barriers, developing targeted delivery systems for alkalinizing agents will be essential for advancing this therapy toward clinical application.

TRPA1 has been reported as a primary alkaline-sensitive TRP isoform [5,23]. Since an alkaline environment (pH 8.1) induced significant cell death in melanoma cells, we investigated whether TRPA1 activator induces cell death in melanoma cells. Although TRPA1 has been extensively characterized as a chemosensor of noxious stimuli in sensory neurons, its contribution to cancer cell physiology remains largely unexplored. The expression of the TRPA1 protein has been reported to increase in several types of solid tumors [38]. The increase in TRPA1 protein expression has also been demonstrated in melanoma cell lines, but the functional role has been unclear [39,40,41]. Our findings highlight a novel functional role for TRPA1 in melanoma. Here, we showed that melanoma cells are sensitive to the electrophilic TRPA1 agonist AITC and evoke a sustained increase in [Ca^2+^]_i_. In addition, they were sensitive to the TRPA1 inhibitor HC-030031 and removal of [Ca^2+^]_o_. Transient increase in [Ca^2+^]_i_ in cancer cells has been known to stimulate cell proliferation and survival, while long-lasting elevations in [Ca^2+^]_i_ reportedly induce cell death [38,42,43]. Importantly, TRPA1 inhibitor HC-030031 and removal of [Ca^2+^]_o_ reduced AITC-induced necrosis, supporting the causal role of sustained Ca^2+^ influx in TRPAptosis.

On the other hand, previous reports suggest that TRPA1 act as a polymodal receptor whose gating behavior is highly dependent on the biological context. Han et al. [44] reported the extracellular binding of miRNA-711 to TRPA1 as a mechanism for pruritus, rather than proton-mediated gating. Faris et al. [38] reported that TRPA1-mediated calcium entry and mitochondrial dysfunction triggered by reactive oxygen species (ROS) in colorectal carcinoma, rather than by extracellular acidity. As de la Roche et al. [45] reported, the functional property in the human TRPA1 ortholog is influenced by residues within the transmembrane domains (TM5 and TM6) that are poorly conserved in rodents, a more recent study specifically comparing canine, mouse, and human TRPA1 demonstrates that the amino acid sequences of the TM5 domain are highly conserved between canines and humans [46]. This structural conservation suggests that the fundamental gating properties and chemical sensitivities of canine TRPA1 more closely mirror those of the human ortholog than those of rodent models. These findings support the translational relevance of the canine melanoma model for human disease.

Mechanistically, our pharmacological and genetic analyses revealed that PLD1 plays a critical role in TRPAptosis. Among several RCD pathway inhibitors (apoptosis, necroptosis, and mitochondrial cell death), only the PLD inhibitor, FIPI, significantly attenuated TRPA1-induced cell death, suggesting that PLD activity contributes to TRPAptosis. Moreover, siRNA-mediated knockdown of PLD1, but not of PLD2, PLD3, or NAPE-PLD and suppressed necrosis in response to TRPA1 activation. Given that PLD generates phosphatidic acid, which can stimulate Ca^2+^ mobilization, our results suggest a positive feedback loop between PLD1 and TRPA1–Ca^2+^ signaling in driving melanoma cell necrosis.

In contrast to plant PLDs, which harbor an N-terminal C2 domain for direct Ca^2+^ coordination, mammalian PLD1 and PLD2 lack this specific structural motif [47]. Therefore, mammalian PLD1 activation is typically regulated by Ca^2+^-sensitive upstream regulators. For instance, classical protein kinase C (cPKC) isoforms, activated by elevated intracellular Ca^2+^, have been shown to mediate PLD1 activation via post-translational modifications [48,49,50]. In this study, we systematically evaluated whether such canonical Ca^2+^-dependent proteins or major kinase pathways bridge the TRPA1-Ca^2+^ influx to PLD1-mediated cell death. We screened a panel of inhibitors targeting calpain [calpain inhibitor II], CaMKII [KN93], pan-PKC [Go6983], and cPKC [Go6976], as well as several protein kinases (ERK1/2 [FR180204], p38 MAPK [SB203586], JNK [SP600125], PI3K [LY294002], and GSK3β [CHIR99021]). Notably, none of these inhibitors attenuated AITC-induced cell death. These results suggest that the TRPA1-mediated sustained increase in [Ca^2+^]_i_ activates PLD1 through a non-canonical mechanism, bypassing traditional kinase-mediated signaling. Further investigations are currently underway in our laboratory to elucidate the intermediate steps.

These observations expand the current understanding of the regulation of cell death in cancer cells. Previous studies have mainly focused on caspase-dependent apoptosis or RIPK/MLKL-mediated necroptosis as the major cell death pathways. Our results indicate that melanoma cells are vulnerable to a distinct necrosis-like program mediated by TRPA1 and PLD1. From a therapeutic perspective, this raises the intriguing possibility that pharmacological activation of TRPA1 or modulation of PLD1 activity could be leveraged as an anti-melanoma strategy. Unlike apoptosis, which is often evaded by tumor cells through mutations in apoptotic regulators, necrosis-like cell death triggered by TRPA1 may bypass conventional resistance mechanisms.

While our findings provide a compelling proof-of-concept for TRPA1 over-activation as a tumor-suppressive strategy, several critical limitations must be addressed before considering clinical translation. First, the present study relied exclusively on in vitro experimental models. While these models effectively demonstrate the TRPA1-Ca^2+^-PLD1 signaling axis, the cell models cannot fully recapitulate the systemic physiological responses or the complex pharmacokinetics within a living organism. Therefore, further validation using preclinical in vivo models is essential to confirm the anti-tumor efficacy of TRPA1 agonists in a more clinically relevant context.

Furthermore, the potential for systemic toxicity remains a significant concern. TRPA1 is known to be widely expressed in various normal tissues, most notably in sensory neurons, where its activation is associated with pain, inflammation, and respiratory irritation. Consequently, the systemic administration of potent TRPA1 agonists could lead to prohibitive off-target effects. To mitigate these risks, future therapeutic strategies must prioritize the development of localized delivery systems or tumor-specific agonists that exploit the high TRPA1 density observed in melanoma cells compared to surrounding normal tissues. Defining the precise ‘therapeutic window’—the dosage range where TRPAptosis is selectively induced in cancer cells without triggering intolerable sensory or systemic toxicity—will be the pivotal next step in determining the clinical viability of this approach.

## 5. Conclusions

We identified TRPA1 as a novel regulator of necrosis-like cell death in melanoma cells and established a functional role for PLD1 and Ca^2+^ signaling in this process. Our findings not only expand the repertoire of regulated cell death pathways but also suggest TRPA1 activation as a unique therapeutic strategy in melanoma.

## Figures and Tables

**Table 1:** Primers used for RT-qPCR.

Gene Symbol	Forward (F) or Reverse (R)	Primer Sequences (5′-3′)	Gene Bank ID
*PLD1*	F	TGTATTCAGAACTGTGAACCCAGGA	XM_022414244.1
	R	AGAGCATTGATTGTGAGGCAGAGA	
*PLD2*	F	CAAAGTGGGCGATGAGATTGTG	XM_005620316.3
	R	GCAGAATGGCCTGGATGGA	
*PLD3*	F	TCTACATCGGCAGTGCCAACA	XM_005616563.3
	R	CCCAGGTACCAATAGGCCTCAA	
*NAPE-PLD*	F	GACACTGGCTACTGCTCAGCTTTC	XM_005630979.3
	R	GGGTCCACGTGCTGGTATTTC	
*TBP*	F	ATGGTGTGTACGGGAGCCAAG	XM_863452
	R	ACTGTTGGTGGGTCAGCACAAG	

**Table 2:** siRNA sequences.

Gene	siRNA Sequences	Gene Bank ID
*PLD1*	CACAACAGGGAGUGAGAAUdTdT	XM_022414244.1
*PLD2*	CUCUGAACCUGCUGCCACAdTdT	XM_005620316.3
*PLD3*	CUAUGACACCCGCUAUAAUdTdT	XM_005616563.3
*NAPE-PLD*	GAGGUGAGUGACUGUGAUAdTdT	XM_005630979.3

**Table 3:** Inhibitors.

Inhibitor	Target	Vendor	Dose (μM)	Pretreatment (h)
FIPI	PLD	Sigma-Aldrich (St. Louis, MO, USA)	50	1
FR180204	ERK	Sigma-Aldrich	50	1
SB239063	p-38	Sigma-Aldrich	20	1
SP600125	JNK	Sigma-Aldrich	10	1
LY294002	pan-PI3K	Cell Signaling Technology (Danvers, MA, USA)	50	1
Calpain inhibitor II	Calpain	Cayman (Ann Arbor, MI, USA)	80	1
KN93	CAMKII	Sigma-Aldrich	10	1
GO6983	pan-PKC	Selleck (Houston, TX, USA)	10	1
GO6976	Classical PKC	Selleck	10	1
CHIR99021	GSK-3	Selleck	10	1
Z-VAD-FMK	Caspase	Selleck	50	1
Necrostatin-1	RIPK	Cayman	20	1
IM-54	Oxidative stress	Cayman	20	1

## Data Availability

The data supporting this study are available within the article and its Supplementary Materials. Further inquiries can be directed to the corresponding author.

## Supplementary Materials

The following supporting information can be downloaded at: https://www.mdpi.com/article/10.3390/cells15090760/s1, Figure S1: The mRNA expression of TRPA1 in human skin cutaneous melanoma (SKCM, red box) and normal melanocyte (grey box) using TCGA and GTEx. TRPA1 mRNA expression increased in human SKCM. Figure S2: Validation of siRNA-mediated PLD1 knockdown in canine melanoma cells. Western blot analysis shows that the protein expression of PLD1 was successfully reduced in canine melanoma cells following transfection with PLD1-targeted siRNA. β-actin was used as a loading control to ensure equal protein loading.

## References

[1] Suwabe Y., Nakano R., Namba S., Yachiku N., Kuji M., Sugimura M., Kitanaka N., Kitanaka T., Konno T., Sugiya H. (2021). Involvement of GLUT1 and GLUT3 in the growth of canine melanoma cells. PLoS ONE.

[2] Zhang B., Vogelzang A., Miyajima M., Sugiura Y., Wu Y., Chamoto K., Nakano R., Hatae R., Menzies R.J., Sonomura K. (2021). B cell-derived GABA elicits IL-10+ macrophages to limit anti-tumour immunity. Nature.

[3] Xiao H., Li T.K., Yang J.M., Liu L.F. (2003). Acidic pH induces topoisomerase II-mediated DNA damage. Proc. Natl. Acad. Sci. USA.

[4] Morita T., Nagaki T., Fukuda I., Okumura K. (1992). Clastogenicity of low pH to various cultured mammalian cells. Mutat. Res..

[5] Fujita F., Uchida K., Moriyama T., Shima A., Shibasaki K., Inada H., Sokabe T., Tominaga M. (2008). Intracellular alkalization causes pain sensation through activation of TRPA1 in mice. J. Clin. Investig..

[6] Kimura M., Sase T., Higashikawa A., Sato M., Sato T., Tazaki M., Shibukawa Y. (2016). High pH-Sensitive TRPA1 Activation in Odontoblasts Regulates Mineralization. J. Dent. Res..

[7] Muramatsu T., Kashiwagi S., Ishizuka H., Matsuura Y., Furusawa M., Kimura M., Shibukawa Y. (2019). Alkaline extracellular conditions promote the proliferation and mineralization of a human cementoblast cell line. Int. Endod. J..

[8] Takahashi N., Chen H.Y., Harris I.S., Stover D.G., Selfors L.M., Bronson R.T., Deraedt T., Cichowski K., Welm A.L., Mori Y. (2018). Cancer Cells Co-opt the Neuronal Redox-Sensing Channel TRPA1 to Promote Oxidative-Stress Tolerance. Cancer Cell.

[9] Oehler B., Scholze A., Schaefer M., Hill K. (2012). TRPA1 is functionally expressed in melanoma cells but is not critical for impaired proliferation caused by allyl isothiocyanate or cinnamaldehyde. Naunyn Schmiedeberg’s Arch. Pharmacol..

[10] De Logu F., Souza Monteiro de Araujo D., Ugolini F., Iannone L.F., Vannucchi M., Portelli F., Landini L., Titiz M., De Giorgi V., Geppetti P. (2021). The TRPA1 Channel Amplifies the Oxidative Stress Signal in Melanoma. Cells.

[11] Tang D., Kang R., Berghe T.V., Vandenabeele P., Kroemer G. (2019). The molecular machinery of regulated cell death. Cell Res..

[12] Kitanaka N., Nakano R., Kitanaka T., Namba S., Konno T., Nakayama T., Sugiya H. (2018). NF-κB p65 and p105 implicate in interleukin 1β-mediated COX-2 expression in melanoma cells. PLoS ONE.

[13] Nunomura J., Nakano R., Naruke A., Suwabe Y., Nakano M., Yachiku N., Kuji M., Sugimura M., Namba S., Kitanaka T. (2022). Interleukin-1β triggers matrix metalloprotease-3 expression through p65/RelA activation in melanoma cells. PLoS ONE.

[14] Nakano R., Kitanaka T., Namba S., Kitanaka N., Sato M., Shibukawa Y., Masuhiro Y., Kano K., Matsumoto T., Sugiya H. (2020). All-trans retinoic acid induces reprogramming of canine dedifferentiated cells into neuron-like cells. PLoS ONE.

[15] Nakano R., Kitanaka T., Namba S., Kitanaka N., Suwabe Y., Konno T., Yamazaki J., Nakayama T., Sugiya H. (2020). Non-Transcriptional and Translational Function of Canonical NF-*κ*B Signaling in Activating ERK1/2 in IL-1*β*-Induced COX-2 Expression in Synovial Fibroblasts. Front. Immunol..

[16] Naruke A., Nakano R., Nunomura J., Suwabe Y., Nakano M., Namba S., Kitanaka T., Kitanaka N., Sugiya H., Nakayama T. (2021). Tpl2 contributes to IL-1β-induced IL-8 expression via ERK1/2 activation in canine dermal fibroblasts. PLoS ONE.

[17] Mizuno M., Nakano R., Nose S., Matsumura M., Nii Y., Kurogochi K., Sugiya H., Uechi M. (2022). Canonical NF-κB p65, but Not p105, Contributes to IL-1β-Induced IL-8 Expression in Cardiac Fibroblasts. Front. Immunol..

[18] Muromachi K., Nakano R., Fujita-Yoshigaki J., Sugiya H., Tani-Ishii N. (2023). BMP-1-induced GBA1 nuclear accumulation provokes CCN2 mRNA expression via importin-β-mediated nucleocytoplasmic pathway. J. Cell Commun. Signal..

[19] Nakano R., Edamura K., Sugiya H., Narita T., Okabayashi K., Moritomo T., Teshima K., Asano K., Nakayama T. (2013). Evaluation of mRNA expression levels and electrophysiological function of neuron-like cells derived from canine bone marrow stromal cells. Am. J. Vet. Res..

[20] Nakano R., Kitanaka T., Namba S., Kitanaka N., Sugiya H. (2018). Protein kinase Cε regulates nuclear translocation of extracellular signal-regulated kinase, which contributes to bradykinin-induced cyclooxygenase-2 expression. Sci. Rep..

[21] Kimura M., Nomura S., Ouchi T., Kurashima R., Nakano R., Sekiya H., Kuroda H., Kono K., Shibukawa Y. (2025). Intracellular cAMP signaling-induced Ca^2+^ influx mediated by calcium homeostasis modulator 1 (CALHM1) in human odontoblasts. Pflug. Arch..

[22] Kang Y.J., Pan L., Liu Y., Rong Z., Liu J., Liu F. (2025). GEPIA3: Enhanced drug sensitivity and interaction network analysis for cancer research. Nucleic Acids Res..

[23] Laursen W.J., Anderson E.O., Hoffstaetter L.J., Bagriantsev S.N., Gracheva E.O. (2015). Species-specific temperature sensitivity of TRPA1. Temperature.

[24] Uchida K., Miura Y., Nagai M., Tominaga M. (2012). Isothiocyanates from *Wasabia japonica* activate transient receptor potential ankyrin 1 channel. Chem. Senses.

[25] Shlomovitz I., Speir M., Gerlic M. (2019). Flipping the dogma—Phosphatidylserine in non-apoptotic cell death. Cell Commun. Signal..

[26] Wada T., Penninger J.M. (2004). Mitogen-activated protein kinases in apoptosis regulation. Oncogene.

[27] Walter M., Tepel M., Nofer J.R., Neusser M., Assmann G., Zidek W. (2000). Involvement of phospholipase D in store-operated calcium influx in vascular smooth muscle cells. FEBS Lett..

[28] Jacobs K.M., Bhave S.R., Ferraro D.J., Jaboin J.J., Hallahan D.E., Thotala D. (2012). GSK-3β: A Bifunctional Role in Cell Death Pathways. Int. J. Cell Biol..

[29] Dodo K., Shimizu T., Sasamori J., Aihara K., Terayama N., Nakao S., Iuchi K., Takahashi M., Sodeoka M. (2018). Indolylmaleimide Derivative IM-17 Shows Cardioprotective Effects in Ischemia-Reperfusion Injury. ACS Med. Chem. Lett..

[30] Araki S., Osuka K., Takata T., Tsuchiya Y., Watanabe Y. (2020). Coordination between Calcium/Calmodulin-Dependent Protein Kinase II and Neuronal Nitric Oxide Synthase in Neurons. Int. J. Mol. Sci..

[31] Kaleli H.N., Ozer E., Kaya V.O., Kutlu O. (2020). Protein Kinase C Isozymes and Autophagy during Neurodegenerative Disease Progression. Cells.

[32] Zhang M., Wang G., Peng T. (2021). Calpain-Mediated Mitochondrial Damage: An Emerging Mechanism Contributing to Cardiac Disease. Cells.

[33] Ecker V., Stumpf M., Brandmeier L., Neumayer T., Pfeuffer L., Engleitner T., Ringshausen I., Nelson N., Jücker M., Wanninger S. (2021). Targeted PI3K/AKT-hyperactivation induces cell death in chronic lymphocytic leukemia. Nat. Commun..

[34] Degterev A., Huang Z., Boyce M., Li Y., Jagtap P., Mizushima N., Cuny G.D., Mitchison T.J., Moskowitz M.A., Yuan J. (2005). Chemical inhibitor of nonapoptotic cell death with therapeutic potential for ischemic brain injury. Nat. Chem. Biol..

[35] Dodo K., Katoh M., Shimizu T., Takahashi M., Sodeoka M. (2005). Inhibition of hydrogen peroxide-induced necrotic cell death with 3-amino-2-indolylmaleimide derivatives. Bioorg. Med. Chem. Lett..

[36] Brown H.A., Thomas P.G., Lindsley C.W. (2017). Targeting phospholipase D in cancer, infection and neurodegenerative disorders. Nat. Rev. Drug Discov..

[37] Robey I.F., Baggett B.K., Kirkpatrick N.D., Roe D.J., Dosescu J., Sloane B.F., Hashim A.I., Morse D.L., Raghunand N., Gatenby R.A. (2009). Bicarbonate increases tumor pH and inhibits spontaneous metastases. Cancer Res..

[38] Faris P., Rumolo A., Pellavio G., Tanzi M., Vismara M., Berra-Romani R., Gerbino A., Corallo S., Pedrazzoli P., Laforenza U. (2023). Transient receptor potential ankyrin 1 (TRPA1) mediates reactive oxygen species-induced Ca^2+^ entry, mitochondrial dysfunction, and caspase-3/7 activation in primary cultures of metastatic colorectal carcinoma cells. Cell Death Discov..

[39] Maglie R., Souza Monteiro de Araujo D., Antiga E., Geppetti P., Nassini R., De Logu F. (2021). The Role of TRPA1 in Skin Physiology and Pathology. Int. J. Mol. Sci..

[40] Zheng J., Liu F., Du S., Li M., Wu T., Tan X., Cheng W. (2019). Mechanism for Regulation of Melanoma Cell Death via Activation of Thermo-TRPV4 and TRPV2. J. Oncol..

[41] Brusco I., Li Puma S., Chiepe K.B., da Silva Brum E., de David Antoniazzi C.T., de Almeida A.S., Camponogara C., Silva C.R., De Logu F., de Andrade V.M. (2020). Dacarbazine alone or associated with melanoma-bearing cancer pain model induces painful hypersensitivity by TRPA1 activation in mice. Int. J. Cancer.

[42] Giorgi C., Baldassari F., Bononi A., Bonora M., De Marchi E., Marchi S., Missiroli S., Patergnani S., Rimessi A., Suski J.M. (2012). Mitochondrial Ca^2+^ and apoptosis. Cell Calcium.

[43] Rosa N., Ivanova H., Wagner L.E., Kale J., La Rovere R., Welkenhuyzen K., Louros N., Karamanou S., Shabardina V., Lemmens I. (2022). Bcl-xL acts as an inhibitor of IP3R channels, thereby antagonizing Ca^2+^-driven apoptosis. Cell Death Differ..

[44] Han Q., Liu D., Convertino M., Wang Z., Jiang C., Kim Y.H., Luo X., Zhang X., Nackley A., Dokholyan N.V. (2018). miRNA-711 Binds and Activates TRPA1 Extracellularly to Evoke Acute and Chronic Pruritus. Neuron.

[45] de la Roche J., Eberhardt M.J., Klinger A.B., Stanslowsky N., Wegner F., Koppert W., Reeh P.W., Lampert A., Fischer M.J., Leffler A. (2013). The molecular basis for species-specific activation of human TRPA1 protein by protons involves poorly conserved residues within trans-membrane domains 5 and 6. J. Biol. Chem..

[46] Yamaguchi T., Uchida K., Yamazaki J. (2023). Canine, mouse, and human transient receptor potential ankyrin 1 (TRPA1) channels show different sensitivity to menthol or cold stimulation. J. Vet. Med. Sci..

[47] Qin C., Wang X. (2002). The Arabidopsis phospholipase D family. Characterization of a calcium-independent and phosphatidylcholine-selective PLD zeta 1 with distinct regulatory domains. Plant Physiol..

[48] Siddiqi A.R., Srajer G.E., Leslie C.C. (2000). Regulation of human PLD1 and PLD2 by calcium and protein kinase C. Biochim. Biophys. Acta.

[49] Hu T., Exton J.H. (2003). Mechanisms of regulation of phospholipase D1 by protein kinase Calpha. J. Biol. Chem..

[50] Park S.Y., Han J.S. (2018). Phospholipase D1 Signaling: Essential Roles in Neural Stem Cell Differentiation. J. Mol. Neurosci..

