# Supplementary figures and images for: Involvement of TRPA1 in Necrosis of Melanoma Cells via Phospholipase D1
Source: Cells. 2026 Apr 23;15(9):760. doi: 10.3390/cells15090760 (PMC13162945; doi:10.3390/cells15090760)

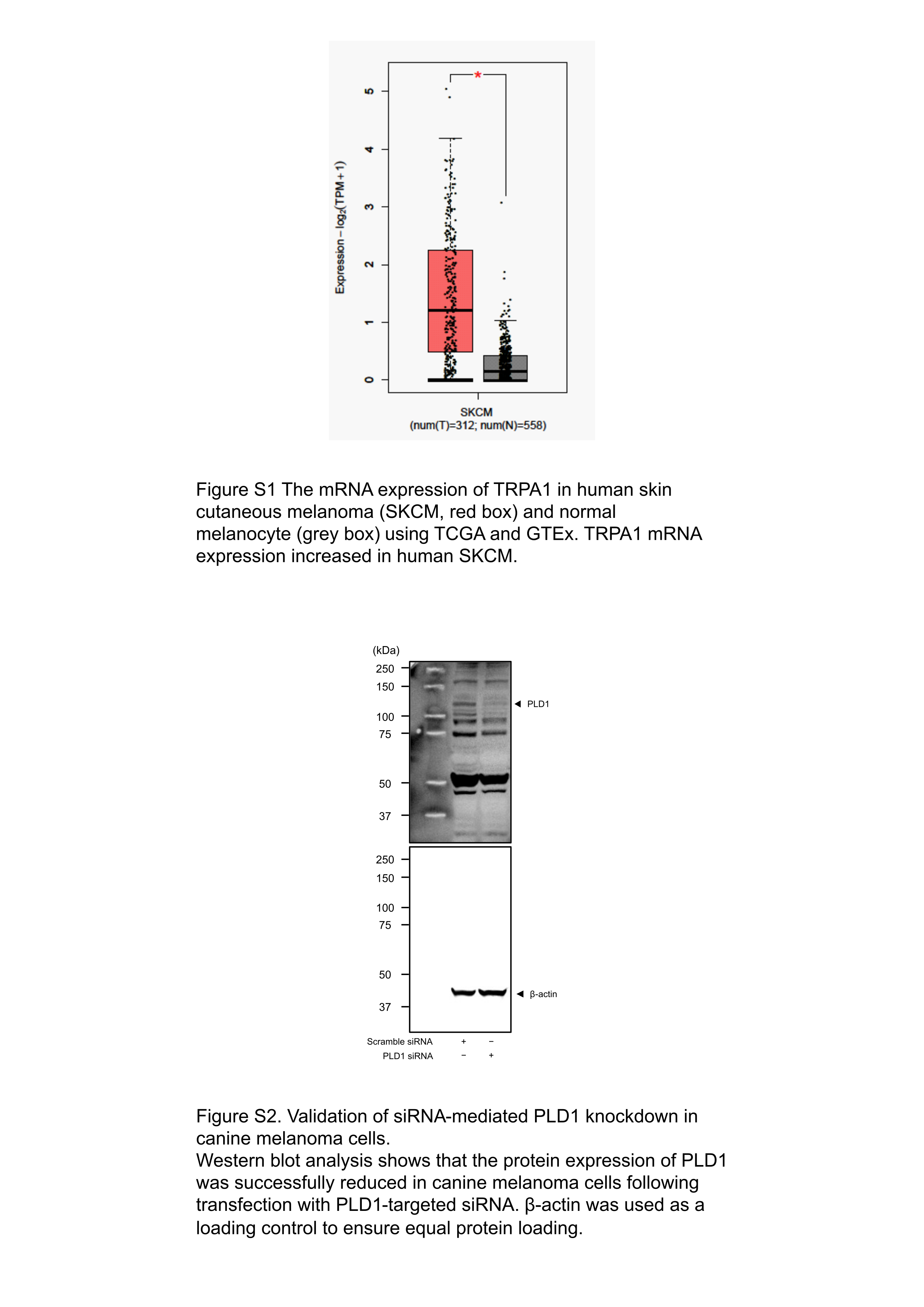

Supplement: Supplementary file 1 [file cells-15-00760-s001.zip › cells-4195934-supplementary.jpg]
